# Feeding or Failing? Rethinking Nutrition Therapy in Pediatric Critical Care

**DOI:** 10.1007/s13668-026-00785-x

**Published:** 2026-07-22

**Authors:** Cecilia Gállego Suárez, Nora Ponchak, Senthilkumar Sankararaman, Akash Pandey

**Affiliations:** 1 Division of Pediatric Critical Care Medicine, Department of Pediatrics, University Hospitals Cleveland Medical Center, Case Western Reserve University School of Medicine, 11100 Euclid Ave, mailstop 6010, suite 3100, Cleveland, OH 44106 USA; 2 Department of Clinical Nutrition, University Hospitals Cleveland Medical Center, Cleveland, OH USA; 3 Division of Pediatric Gastroenterology, Hepatology and Nutrition, Cleveland Clinic Children’s Hospital, Cleveland, OH USA; 4 Division of Pediatric Gastroenterology, Hepatology and Nutrition, Center for Digestive Health & Nutrition, Arnold Palmer Hospital for Children, Orlando, FL USA

**Keywords:** Pediatric critical illness, Malnutrition, Enteral nutrition, Parenteral nutrition, Rehabilitation

## Abstract

**Purpose of Review:**

Malnutrition, whether pre-existing or acquired in the ICU, is common in critically ill children. This narrative review synthesizes current evidence on nutritional assessment and therapy, highlighting how illness phase, underlying disease, and medical interventions influence energy expenditure and nutrient requirements.

**Recent Findings:**

International guidelines recommend comprehensive nutritional assessment and early initiation of enteral nutrition (EN) for all patients admitted to the Pediatric ICU. However, EN is frequently underutilized or insufficient to meet metabolic demands and sometimes contraindicated. Barriers to delivery persist, and parenteral nutrition (PN) may be required when EN is not feasible or adequate.

**Summary:**

Nutrition plays a crucial yet complex role in the care of critically ill children. Optimizing individualized nutrition strategies, including timely EN and appropriate PN use, is essential to improve clinical outcomes, support recovery, and enhance long-term rehabilitation, while highlighting the need for improved implementation and future research.

## Introduction

Malnutrition, a complex global health challenge, disproportionately affects children worldwide and remains prevalent across all socioeconomic groups. It encompasses various forms of undernutrition (wasting, stunting, and underweight), as well as overweight, obesity, and micronutrient deficiencies. In 2022, the World Health Organization reported alarming statistics: 149 million children under the age of 5 were stunted, 45 million had wasting, and 37 million had overweight or obesity. It is estimated that approximately half of the deaths among children under the age of five in low- and middle-income countries are linked to malnutrition. The situation is equally concerning for children aged 5 to 19 years, with approximately 188 million having underweight and 391 million having overweight or obesity. For the first time in 2025, overweight and obesity have become more prevalent than underweight in this age group [1, 2].

Micronutrient deficiencies are highly prevalent among children and adolescents, affecting approximately one third to half of them globally and can have significant consequences, including immunological dysfunction, increased risk for infection, anemia, cardiovascular disease, bleeding, neuromuscular weakness, thyroid dysfunction, cognitive impairment and growth faltering [3–7].

Malnutrition is a prevalent comorbidity in critically ill children. A recent systematic review and meta-analysis estimated that 37% of this population experience undernutrition [8], while approximately 11–14% have overweight and 14–15% have obesity worldwide [9, 10]. Undernutrition is associated with increased mortality, longer ICU and hospital length of stay (LOS), hospital-acquired infections, fluid overload, delayed wound healing and longer duration of mechanical ventilation. Obesity has been associated with increased ICU LOS, hospital acquired infections and higher utilization of extracorporeal support (Continuous Renal Replacement Therapy (CRRT) and Extracorporeal Membrane Oxygenation (ECMO)), although its impact on mortality is inconclusive [11–17].

Nutrition plays a crucial yet complex role in the care of critically ill children. In this narrative review, we examine current approaches to nutritional assessment and management, identify key barriers to the delivery of optimal nutrition therapy, and highlight implications for improving long-term outcomes.

## Nutritional Assessment in Critically Ill Children

Current guidelines from the Society of Critical Care Medicine (SCCM), the American Society for Parenteral and Enteral Nutrition (ASPEN) [18], and the European Society of Pediatric and Neonatal Intensive Care (ESPNIC) [19] recommend that all children admitted to the Pediatric Intensive Care Unit (PICU) undergo a comprehensive nutritional assessment at or within the first 48 h of admission to identify pre-existing malnutrition or risk thereof.

### History and Screening Tools

A nutrition-focused history, including weight changes, dietary intake and quality, history of prematurity or low birth weight, comorbidities that affect metabolic needs or gastrointestinal function, prior or concurrent enteral access, and medications or supplements utilization, should be obtained. Several nutrition screening tools exist (STRONGkids, Pediatric Yorkhill Malnutrition Score, STAMP) and can help identify children at risk for nutrition deterioration during PICU admission, nevertheless, their accuracy is variable and none have been validated for the critically ill pediatric population [18–21].

### Physical Exam

Admission anthropometrics may be inaccurate and/or incomplete upon PICU admission due to several factors such as clinical instability, strict immobilization, presence of medical equipment or extracorporeal support circuits, fluid overload, or severe dehydration. When feasible, weight, height/length, and head circumference (in children < 36 months of age), should be measured on admission. Age- and sex-specific weight, height, and BMI percentiles and Z-scores are utilized to evaluate baseline nutritional status and changes reflective of malnutrition [22–24]. Mid-upper arm circumference (MUAC) measures muscle and subcutaneous fat mass and is unaffected by fluid status, and in the absence of anthropometrics, it can be utilized alone as a diagnostic criterion for malnutrition [25, 26].

### Estimating Energy Expenditure

Estimating energy expenditure in critically ill children is particularly challenging due to the wide range of factors that influence it. These include differences in age, weight, body composition, growth, the varying severity of illness and metabolic response, and the dynamic changes in energy requirements that occur during critical illness [18, 27–29]. Critical illness is characterized by an increase in resting energy expenditure (REE), but in the early phase or under certain circumstances, it can be normal or decreased [29–31]. REE is reduced by hypothermia, deep sedation, neuromuscular blockade, and severe hypothyroidism while fevers, burns, and agitation increase it[18]. Indirect Calorimetry (IC) is considered the gold standard to objectively evaluate energy needs, but its availability is limited and it is unreliable and inaccurate in the presence of air leaks (around the endotracheal tube, tracheostomy, or chest tubes), high FiO_2_ (> 60%), extracorporeal CO_2_ removal on ECMO, and dialysis including CRRT [18, 29, 32].

When IC is unavailable, the Schofield or WHO predictive equations (without counting stress factors) can be utilized, however, these equations over or underestimate REE by +/- 10% and can lead to unintended overfeeding or underfeeding [18, 19, 29, 33].

### Biochemical Evaluation

No single biochemical marker exists to diagnose malnutrition in children. Serum proteins such as pre-albumin and albumin are affected by the inflammatory response of critical illness and do not reliably reflect nutritional status [34, 35]. Accordingly, current guidelines advice against using laboratory values as primary diagnostic criteria for malnutrition [21, 36]. Rather, laboratory values - including protein levels, electrolytes, and micronutrients - should be interpreted in the context of end-organ function, the inflammatory response and overall physiology, while the diagnosis of malnutrition should rely on anthropometric measurements and clinical history.

## Nutrition Principles in Pediatric Critical Illness

The metabolic response in critical illness driven by complex immunological and neuro-hormonal mechanisms result in a catabolic state characterized by insulin and growth hormone resistance, mitochondrial dysfunction, mobilization of endogenous substrates with increased glycolysis and glycogen storage depletion, accelerated protein breakdown, utilization of amino acids and glycerol for hepatic gluconeogenesis, and impaired fatty acid oxidation [30, 31, 37]. This leads to a negative nitrogen balance, stress hyperglycemia, loss of lean body mass, and impaired nutrient utilization.

Energy and nutrient needs vary across developmental stages. In critically ill children, a minimum protein intake of 1.5 g/kg/day is currently recommended to promote a positive nitrogen balance and lean muscle mass accretion, but infants and younger children might have higher requirements up to 2.0–3.0 g/kg/day[18, 34]. Once the energy and protein requirements have been determined, the carbohydrate and fat requirements can be calculated. Carbohydrates are the primary energy substrate for the brain and many tissues with high metabolic demands, and its provision should be balanced to minimize further protein catabolism while avoiding hyperglycemia and excessive CO_2_ production [38, 39]. Lipids should be administered not only as an energy substrate but also for their essential role in biochemical processes that support cellular metabolism. They can be delivered via enteral or parenteral routes (intravenous fat emulsions - IVFEs) and dose should not exceed the rate of lipid clearance [40, 41]. Children are particularly susceptible to essential fatty acid deficiencies during critical illness due to limited adipose tissue stores coupled with increased lipid metabolism.

In terms of micronutrients (minerals and vitamins), limited research exists in this patient population to suggest supplementation beyond current dietary reference standards [42, 43]. Additionally, the acute systemic inflammatory response affects micronutrient concentrations in plasma, with most decreasing proportionally with the severity of inflammation. Studies have shown vitamins A, B6, C, and D, and selenium plasma concentrations are affected even at mildly elevated CRP levels (5–10 mg/L), and zinc levels to be unreliable if CRP levels are above 20 mg/L[44, 45]. Therefore, if measured, levels should be interpreted cautiously. Supplementation is warranted in patients with high metabolic stress, underlying malnutrition and risk for refeeding syndrome, and those requiring CRRT.

Evidence suggests a phase-adapted approach to provide nutrition during critical illness, with a goal of delivering 60–70% of protein and energy requirements during the acute catabolic phase (by the end of the first week of PICU stay). Subsequently, gradual advancements are made to meet higher protein and energy needs during the hypermetabolic phase observed in the stabilization and recovery periods [18, 19, 46]. Table 1 provides a concise summary of recommendations outlined by ASPEN/SCCM and ESPNIC guidelines. Children recovering from critical illness need to consume at least twice their REE to facilitate catch-up growth and regeneration of lean muscle mass, with even higher needs if significant physical activity is incorporated into their rehabilitation[47]. Certain conditions, such as burns, traumatic brain injury, and congenital heart defects repairs, are associated with higher demands due to chronic hypermetabolic state.

**Table 1:** Summary of recommendations by SCCM / ASPEN [18] and ESPNIC [19] guidelines on nutrition in critically ill children

Upon admission, obtain anthropometric measurements and assess nutritional status.
Determine energy requirements through indirect calorimetry (IC). If IC is unavailable or unfeasible, utilize Schofield or World Health Organization equations without stress factors.
Achieve at least two-thirds of the estimated energy requirements by end of first week of PICU admission.
Minimum protein intake of 1.5 g/kg/day, early to promote positive nitrogen balance.
Enteral nutrition (EN) as the preferred mode of nutrition delivery with early initiation (within 24–48 h of admission) unless contraindicated and increase in stepwise manner until at goal. Gastric feeding preferred unless not tolerated or high risk for aspiration.
Start early EN in patient with stable hemodynamics on extracorporeal support or vasoactives, or prostaglandin E1 infusion.
Nutrition prescription in the early phase should not exceed resting energy expenditure (REE), increase in the recovery and rehabilitation phases to account for increased energy requirements.
Do not recommend initiation of PN in the first 24 hours of PICU admission and can be delayed until day 7 in children with normal nutritional status and low risk for malnutrition.

*IC* indirect calorimetry, *PICU* Pediatric Intensive Care Unit, *EN* enteral nutrition, *PN* parenteral nutrition

Both overfeeding and underfeeding have negative consequences and risks. Overfeeding is associated with hyperglycemia, hypertriglyceridemia, increased CO_2_ production (which can prolong duration of invasive mechanical ventilation), liver dysfunction and steatosis, hospital acquired infections, anabolic resistance (characterized by decreased muscle protein synthesis due to impaired aminoacid uptake, insulin resistance, and disruption of cellular repair pathways), and longer ICU LOS. Conversely, underfeeding can worsen pre-existing malnutrition and is associated with ICU-acquired weakness, longer duration of invasive mechanical ventilation, ICU and hospital LOS [18, 27, 48–51].

## Medications Affecting Nutrition

Several medications essential for treating critically ill children have adverse effects on the gastrointestinal system and nutrition. Opioids used for analgesia and sedation affect gastrointestinal motility, decrease pancreatic, biliary, and intestinal secretions affecting nutrient absorption, and are known to cause constipation, nausea, emesis, and ileus [52, 53]. Proton pump and H2 inhibitors commonly used for stress ulcer prophylaxis suppress gastric acid secretion affecting protein and micronutrients absorption, affect the microbiome, and are associated with increased risk of hospital-acquired pneumonia and *C. Difficile* infections [54–56]. Broad-spectrum antibiotics disrupt the gut microbiome affecting nutrient absorption and metabolism [57–59]. Enteral nutrition (EN) is often delayed in children requiring vasoactives/inotropes due to concerns for bowel ischemia or feeding intolerance. Nevertheless, on the basis of observational studies demonstrating tolerance and no adverse effects, international guidelines support early EN initiation if hemodynamic stability and adequate perfusion have been achieved on stable or weaning doses of vasoactives [18, 19, 60]. Equally, the use of non-depolarizing neuromuscular blockade agents is not a contraindication for EN as they do not affect the smooth muscle in the gastrointestinal tract [61, 62].

## Disease Specific Nutritional Care

### Respiratory Failure

Children with acute respiratory failure frequently experience poor oral intake before and during their hospitalization and are at high risk of ICU-acquired malnutrition [63]. Adequate early enteral nutrition is associated with decreased mortality, ICU LOS, and duration of invasive mechanical ventilation (IMV) [18, 64–66]. International Guidelines in pediatric acute respiratory distress syndrome (pARDS) recommend initiating EN in the first 72 hours, with adequate protein and energy delivery to maintain growth and meet metabolic demands [67]. To date, the evidence on the safety and optimal delivery of nutrition in children receiving non-invasive respiratory support, such as high-flow nasal cannula or non-invasive positive pressure ventilation, is limited to observational studies. These studies support the safety of gastric feeds and their early initiation, with aspiration events being rare [68–71].

### Heart Failure (HF)

Heart failure from congenital heart defects or cardiomyopathies is associated with significantly increased energy expenditure and a high prevalence of malnutrition [72, 73] and is independently associated with increased mortality, prolonged ICU and hospital LOS, and need for IMV [74]. Impaired systemic circulation seen in decompensated heart failure or single ventricle physiology increases the risk for feeding intolerance, impaired absorption, and bowel ischemia. Children with HF need a nutrient-dense and fluid-restricted approach to nutrition. They may also require supplemental enteral or parenteral nutrition (PN) and micronutrient supplementation [73, 75].

### Sepsis

In pediatric sepsis, enteral nutrition can help preserve the gut mucosal integrity and microbiome, decrease bacterial translocation, and modulate the inflammatory response [76]. The Surviving Sepsis Campaign Guidelines in Pediatric Sepsis support enteral nutrition once hemodynamic stability has been achieved on stable or weaning doses of vasoactives, withholding parenteral nutrition in the first 7 days of ICU admission, and recommend against the routine use of selenium, glutamine, zinc, vitamin C, D and thiamine for the treatment of sepsis or septic shock in children [60].

### Traumatic Brain Injury (TBI)

Initiation of enteral nutrition within 72 hours from injury is recommended to improve survival and outcomes [77, 78]. Special attention should be made to maintain normoglycemia, euvolemia, and adequate serum sodium levels according to neuro-resuscitation goals. Nutrition prescriptions should meet the energy and protein requirements, which vary depending on the phase (acute vs. rehabilitation) and the exposure to sedation or neuromuscular blockade. Severe traumatic brain injury (TBI) can cause refractory intracranial hypertension, necessitating the initiation of barbiturate (pentobarbital) infusion [77]. This can lead to severe ileus, making parenteral nutrition the sole viable option for nutrition.

### Acute Kidney Injury (AKI)

Critically ill children with AKI may have increased prevalence of nutritional deficits within the initial five days of PICU stay [79]. If feasible, oral feeding is the preferred mode of feeding, and early introduction (within 48 hours of admission) of EN (either supplemental or exclusive) should be considered based on recommendations from the Pediatric Renal Nutrition Taskforce [80]. Expressed breast milk and/or age-appropriate low potassium/phosphorus renal-specific formulas may provide adequate energy, protein, and electrolytes. Due to fluid restrictions in children with AKI, protein- and energy-dense formulas can be utilized to improve nutrient delivery, with caloric needs of approximately 100–130% of REE. Importantly, protein prescription should not be compromised to lower blood urea nitrogen levels or postpone renal replacement therapy [80]. Frequent monitoring of acid balance/electrolytes and restriction of potassium, phosphorus, and sodium is important in this patient population.

### Liver Failure

Children with acute liver failure are often catabolic and have higher energy needs than their baseline [81]. Malnutrition is widely prevalent in these patients and worsens their outcomes. As in other critically ill scenarios, EN is preferred over PN in acute liver failure. Adequate protein intake (~ 1–1.5 g/kg/day) can be safely administered as EN while avoiding hyperammonemia. Maintaining normoglycemia and euvolemia can be challenging and should be aggressively managed [82, 83]. Due to impaired gluconeogenesis and glycogenolysis, these patients often develop hypoglycemia requiring high glucose infusion rates to maintain normoglycemia. Macronutrient composition of EN and PN should be adjusted if an inborn error of metabolism is suspected as the underlying cause of acute liver failure with collaboration and guidance with Genetics and Metabolism specialists [83].

### Acute Severe Pancreatitis

Given the paucity of nutritional literature in severe acute pancreatitis in pediatrics, evidence from adult studies can be carefully extrapolated to children [84, 85]. Once hemodynamically stable, early nutrition (within the first 72 hours of admission) should be prescribed and EN is preferred over PN. EN has been shown to reduce organ failure, pancreatitis-related infectious, and mortality [85, 86]. Gastric feeding is safe and easier to initiate compared to post-pyloric feeds and jejunal feeding can be considered if gastric feeds are not feasible or not tolerated [84, 87].

### Oncologic Disease

In children with oncologic disease, malnutrition is a prevalent co-morbidity and challenges in nutritional management due to disease burden, anorexia, chemotherapy-induced nausea and emesis and mucositis are common factors attributing to nutritional failure. Enteral nutrition has been demonstrated to improve outcomes and is always the preferred method of feeding, unless contraindicated. However, in some cases, patients may require supplemental EN via nasogastric or gastrostomy tubes when they are ill or when oral intake is insufficient [88, 89].

### Burns

Children with severe burns develop a hypermetabolic and catabolic state that persists up to 1 to 3 years after the injury [90, 91]. These patients have higher caloric and protein requirements and should be started on enteral nutrition early (as soon as the first 6–12 hours of ICU admission). Ileus from bowel wall edema is common, nevertheless, enteral nutrition has been associated with decreased mortality, infections, gastrointestinal bleeding and a shorter hospitalization. Overfeeding and underfeeding comes with great risks for these patients and objective estimation of energy needs via IC is recommended. Some micronutrients and trace elements (such as water-soluble vitamins, copper, selenium, zinc) are depleted in the acute phase, and close monitoring and supplementation based on clinical and laboratory criteria are warranted [18, 90, 92, 93].

### Extracorporeal Support

Patients who require ECMO for management of cardiopulmonary failure, or CRRT for renal failure or fluid overload, are at high risk for ICU-acquired malnutrition due to the severity of their illness, increased catabolism, prolonged sedation and immobilization, fluid restrictions, enteral or central venous access limitations. Currently, the evidence on nutrition in pediatric ECMO is limited to observational studies that demonstrate enteral nutrition is preferred and safe, but underutilized [94–96]. The Extracorporeal Life Support Organization (ELSO) Guidelines recommend early enteral nutrition initiation in neonates and children receiving ECMO support, ideally within 48 hours, or sooner if clinically stable, and to consider parenteral nutrition within the first 3–5 days for malnourished children and within 5–7 days for those without malnutrition if enteral nutrition is contraindicated or insufficient to meet goals [97]. ESPNIC also supports early enteral nutrition once hemodynamic stability has been achieved on extracorporeal support [19].

In relation to CRRT, certain macro and micronutrients are lost through the circuit and dialysis treatment. These include amino acids, water-soluble vitamins (B1, B6, B9, and C), and selenium. Consequently, patients undergoing CRRT have increased protein requirements to maintain a positive nitrogen balance and necessitate additional supplementation. Both enteral and parenteral nutrition, or a combination of both, are suitable routes for providing nutrition therapy in these patients. Furthermore, necessary adjustments are made based on native renal function and CRRT prescription [98–102].

## Enteral Nutrition

Both the European and American Pediatric Nutrition Guidelines strongly recommend initiation of enteral nutrition (EN) as soon as feasible if there are no contraindications [18, 19]. Pre-clinical studies and animal experiments have demonstrated that lack of EN results in immediate impairment of gut mucosal barrier with translocation of gut bacteria inciting immunological dysfunction resulting in inflammation [103]. On the other hand, early initiation of EN (within 24–48 hours of ICU admission) improves gastrointestinal mucosal integrity, gut motility, and helps to contain further inflammatory milieu associated with critical illness, potentially improving outcomes. Even low volume trophic feeding may reduce potential infectious complications by improving the gut barrier function [103, 104]. Two recent meta-analysis found that early enteral nutrition (within 48 hours of PICU admission) was associated with decreased mortality [105, 106].

EN should be started at low volume and advanced in a steady and gradual way until the goal is achieved. Several factors such as positive pressure ventilation (invasive and non-invasive), severity of illness, impaired perfusion, use of inotropic/vasoactive medications, NPO (nil per os) for procedures or sedation, and medications affecting GI motility such as opioids may be barriers for early EN [107, 108]. Enteral feeds are also frequently interrupted due to perceived feeding intolerance, procedures or fluid restrictions. A recent meta-analysis involving over 4,000 critically ill children revealed that approximately 53% of them experienced interruptions in EN (enteral nutrition), which was associated with longer ICU length of stay [109].

ESPNIC favors the use of EN in various critical scenarios and recommends the use of feeding protocols to improve the delivery of EN in critically ill children [19]. Among the formulas, polymeric feeds should be used as first choice and peptide-based can be recommended if polymeric feeds are not tolerated or contraindicated. Similarly, gastric feeding is recommended, and post-pyloric feeds are utilized only if gastric feeds are not tolerated or contraindicated (high risk of aspiration). Feeds can be either continuous, or bolus and no differences have been noted in studies. Checking gastric residual volumes to guide feeding tolerance is not recommended and this practice should be discouraged given lack of evidence [18, 19, 104]. There is no role for immunonutrition or pharmaconutrition (the utilization of specialized nutrients to potentially modulate the immunological response) due to lack of evidence supporting a benefit or safety [18, 19, 60].

Given the heterogeneity and presence of confounding factors in the small number of available pediatric studies in evaluating feeding strategies [105], well-designed interventional studies are urgently needed. Similarly, implementation of bedside protocols and quality-improvement initiatives will help reduce time to initiate EN and also will minimize variability in clinical practice.

## Parenteral Nutrition

The approach to PN in critically ill children has evolved substantially, with a shift away from early routine initiation toward a more selective, individualized strategy. During the acute phase of critical illness, children rely on endogenous substrate mobilization —gluconeogenesis, lipolysis, and proteolysis—as part of the metabolic stress response[50]. In this context, PN should be regarded as metabolic support rather than a means of early nutritional repletion. Accordingly, PN should complement, rather than counteract, the physiological stress response, with macronutrients introduced once the acute catabolic phase is resolving.

The landmark PEPaNIC trial, a multicenter randomized controlled study of 1,440 critically ill children, demonstrated that withholding PN for one week resulted in significantly fewer new infections (10.7% vs. 18.5%), shorter PICU stays (6.5 vs. 9.2 days), and reduced duration of mechanical ventilation compared to PN initiated within 24 hours [51]. These benefits were consistent across all subgroups—including children at highest nutritional risk, neonates, and those with varying illness severity. Preplanned follow-up at 2 and 4 years demonstrated improved neurocognitive outcomes including executive function and visual-motor integration in the late-PN group [51, 110, 111]. The possible mechanism associated with worse outcomes from early PN initiation appear to be related to amino-acid administration [50]. Early amino-acid delivery suppresses autophagy and ketogenesis, induces anabolic resistance and iatrogenic hyperglycemia, and triggers epigenetic modifications mediating developmental changes [50, 110]. Early PN also carries refeeding syndrome risk—phosphate shifts, thiamine depletion, and fluid overload—particularly in malnourished children [50].

Current ASPEN/SCCM guidelines recommend delaying supplemental PN until one week after PICU admission for patients with normal baseline nutritional status. PN supplementation is suggested for children unable to receive any enteral nutrition (EN) during the first week and may be considered earlier in severely malnourished patients who cannot advance past low EN volumes [18]. Absolute indications include intestinal failure—anatomic disorders such as short bowel syndrome, mucosal disorders, or neuromuscular disorders—where the gastrointestinal tract cannot support adequate nutrition [18, 112].

When PN is indicated, lipid emulsion selection warrants consideration. Composite lipid emulsions containing fish oil are associated with reduced hepatotoxicity compared to pure soybean oil emulsions, particularly in patients at risk for intestinal failure-associated liver disease [113, 114]. Monitoring should include glucose control, triglyceride levels (reduce lipid if > 250 mg/dL; hold if > 400 mg/dL), electrolytes, and hepatic function [41, 112]. Micronutrients—including phosphate, thiamine, zinc, and selenium—should be provided early to prevent deficiencies and refeeding syndrome, even when macronutrients are withheld [50, 115].

Complications of prolonged PN include risk for central line-associated bloodstream infection, intestinal failure-associated liver disease driven by soybean oil-based emulsions and lack of enteral stimulation, and metabolic bone disease [114]. These risks underscore viewing PN as a bridge to enteral autonomy. Transition to EN should proceed once enteral tolerance reaches approximately 60–80% of nutritional targets [18].

## Post-Discharge Follow Up

After PICU discharge, many children enter a vulnerable phase for nutritional failure. Ongoing inflammation, accumulated energy and protein deficits, ICU-related muscle mass loss, and feeding disruptions during care transitions combine to drive deficits that shape long-term recovery and function [116]. Recent evidence demonstrates that many children fail to meet nutritional requirements after PICU discharge. The ePICUre study found that 36% of children on enteral nutrition exclusively received less than half of their estimated energy and protein requirements on the first day after PICU discharge, and the proportion of children that received less than 75% of their estimated energy needs increased from 17% on day 1 to 33% by day 28 [117].

ICU acquired weakness can significantly impair functional recovery, reinforcing the importance of adequate nutritional support. Early mobilization paired with adequate nutrition, are vital for the recovery and rehabilitation of critically ill children [118, 119].

Structured nutritional follow-up within 1–2 weeks after discharge targets the highest-risk transition period, with continued monitoring recommended for up to 6–12 months following PICU discharge. Table 2 provides an overview of the nutritional follow-up after PICU discharge. Weight, height/length, BMI percentiles and z-scores, and mid-upper arm circumference, should be tracked regularly. Point-of-care ultrasound and bioelectrical impedance analysis are practical and accurate tools that can provide valuable information for assessing body composition. During this high risk period, nutritional adequacy and feeding tolerance should be assessed continuously, and continuation of supplemental EN (+/- enteral access) should be considered when oral intake alone is insufficient to support recovery [116, 117]. Multidisciplinary care—including dietitians, physicians, and therapists—is critical to optimizing outcomes in children with persistent feeding challenges.

**Table 2:** Timeline and approach of nutritional follow up after PICU discharge

Recovery Phase	Timeline	Assessment	Intervention
Early Recovery	0–2 weeks post-discharge	**Anthropometrics**: Weight vs. pre-illness baseline; MUAC. **Feeding**: Tolerance assessment; oral aversion screening. **Micronutrients**: Iron, zinc, vitamin D, thiamine.	**Nutrition**: Energy-dense, protein-enriched diet (≥ 1.5 g/kg/day protein)*. **EN support**: Continue supplemental EN if oral intake < 75% of targets. **Referral**: Feeding therapy for oral aversion.
Intermediate Recovery	2–8 weeks	**Growth**: Growth velocity assessment. **Muscle**: POCUS (quadriceps thickness) or BIA if available. **Function**: Developmental and functional milestones.	**Strategy**: Protein-first repletion; early mobilization. Initiate or continue supplemental EN if growth velocity below pre-illness trajectory. **Activity**: Age-appropriate physical rehabilitation.
Late Recovery	> 8 weeks	**Growth**: Length/height velocity; BMI trajectory. **Development**: Developmental milestones; functional recovery assessment. **Nutrition status**: Body composition trends; micronutrient adequacy.	**Wean**: Supplemental support if oral targets met consistently. **Monitor**: Ongoing surveillance if trajectory remains below baseline. **Refer**: Subspecialty referral (GI/Nutrition, Rehabilitation) if persistent failure to recover.

*EN* enteral nutrition, *GI* gastrointestinal, *MUAC* mid-upper arm circumference, *POCUS* Point-of-care ultrasound, *BIA* Bioelectrical Impedance Analysis, *PICU* pediatric intensive care unit

*Protein targets based on ASPEN recovery phase recommendations. Micronutrient screening priorities derived from post-ICU deficiency prevalence data. Growth velocity should be compared to pre-illness trajectory, not population norms.

## Conclusions

Nutrition plays a crucial role in the care of critically ill children. It is essential to assess the underlying nutritional status of pediatric patients who become critically ill and provide them with adequate and goal-directed nutrition to enhance their chances of survival and recovery. When feasible, early EN initiation and efforts to advance to goal and continue while minimizing interruptions or disruptions should be undertaken. Due to the complexity and various factors influencing nutrition prescriptions, collaboration among physicians, dietitians, nurses, therapists, and pharmacists is vital to optimize nutrition delivery. Adjustments should be made as necessary to ensure optimal nutrient delivery, balanced metabolism, lean muscle mass recovery, and ultimately, healing and rehabilitation during and after ICU discharge.

## Key References

- Mehta NM, Skillman HE, Irving SY, et al. Guidelines for the Provision and Assessment of Nutrition Support Therapy in the Pediatric Critically Ill Patient: Society of Critical Care Medicine and American Society for Parenteral and Enteral Nutrition. *Journal of Parenteral and Enteral Nutrition*. 2017;41(5):706–742. doi:10.1177/0148607117711387.
  - ○ The Society of Critical Care Medicine (SCCM) and the American Society for Parenteral and Enteral Nutrition (ASPEN) guidelines, as well as the European Society of Pediatric and Neonatal Intensive Care (ESPNIC) position statements, provide the most comprehensive and evidence-based recommendations for nutrition support in critically ill children.
- Tume LN, Valla F V., Joosten K, et al. Nutritional support for children during critical illness: European Society of Pediatric and Neonatal Intensive Care (ESPNIC) metabolism, endocrine and nutrition section position statement and clinical recommendations. Intensive Care Med. 2020;46(3):411–425. doi:10.1007/s00134-019-05922-5.
  - ○ The Society of Critical Care Medicine (SCCM) and the American Society for Parenteral and Enteral Nutrition (ASPEN) guidelines, as well as the European Society of Pediatric and Neonatal Intensive Care (ESPNIC) position statements, provide the most comprehensive and evidence-based recommendations for nutrition support in critically ill children.
- Gilbert N, Schalm E, Wollny K, Lee L, Boctor DL, Fenton TR. Early Enteral Nutrition and Clinical Outcomes in Critically Ill Pediatric Populations: A Systematic Review and Meta-Analysis. Crit Care Med. 2026;54(1):129–141. doi:10.1097/CCM.0000000000006859.
  - ○ In this systematic review and meta-analysis, early enteral nutrition was associated with decreased mortality, reduced length of hospital stay, organ dysfunction, and infections in critically ill children. However, significant heterogeneity among studies underscores the need for randomized controlled trials to assess the clinical effects of enteral nutrition in this population.

## Data Availability

No datasets were generated or analysed during the current study.
